# Compulsory Re-Vaccination

**Published:** 1896-08-22

**Authors:** 


### The Hospital

An Institutional Journal of

The Medical Sciences and Hospital Administration, with

AN EXTRA NURSING SUPPLEMENT.

Vol. XX.—No. 517.] [Aug. 22, 1896.

### COMPULSORY RE-VACCINATION.

The Royal Commission on Vaccination have at last finished their labours, and it is intimated that Sir Guyer Hunter and Mr. Jonathan Hutchinson have appended to the Report a memorandum advising that not only vaccination, but also re-vaccination, should be rendered compulsory. This is a development of the subject which demands to be dealt with dispassionately, and from the one standpoint of the highest well-being of the State.

Vaccination is altogether a practical question; compulsory vaccination is a question of practical politics. The State authorities have two pictures to look upon—an unvaccinated population decimated by small-pox, on the one hand; and, on the other, a thoroughly vaccinated population among whom small-pox is unknown. It may be taken for granted that the evidence accumulated by the Commission is such as to justify the conclusion that efficient vaccination and re-vaccination would stamp out small-pox altogether. If that were not so, it is quite certain that Mr. Jonathan Hutchinson would not have assumed the responsibility of advising so serious a new departure as compulsory re-vaccination.

The question which rises first in the mind is this, Will the country submit to compulsory re-vaccination? We think it will if care be taken to show it an adequate reason. Unfortunately, the ideas which prevail on the subject are very crude, even among well educated people. But a little reflection may convince the mind that an operation performed during the first six months of infantile existence could not with any reason be expected to produce such a change of constitution as would survive throughout a whole long lifetime. The baby of a few months old is almost as different in every particular from the adult man as to be a distinct creature. If any one wishes to realise the full measure of the difference, let him compare the mind of the newly-born baby with the mind of the full-grown man. There is probably almost as much difference between the tissues of which the baby's body is composed and those of the man as there is between the minds of the two. At twelve or fourteen years of age the case is entirely different. At that period it is quite easy, as a rule, to say with certainty not only what kind of mind, but also what kind of body and constitution a given person is to have in permanence. The permanent trend has, so to speak, been determined. Any operation now of the vaccination order will almost certainly as a rule be productive of effects of a permanent character. The whole case, then, resolves itself into this: First, does the vaccination of babies protect up to the age of ten or twelve? If it does, then compulsory vaccination is desirable for infants. Secondly, does re-vaccination at twelve or fourteen protect permanently? If it does, then compulsory re-vaccination is desirable at that period. The logic of the case is without a flaw. The very same arguments which justify the compulsory vaccination of infants justify compulsory re-vaccination at a maturer age. Only, if possible, the reasoning applies more urgently to the latter age than to the former.

Compulsion has an ugly sound to a democratic people. But, as a matter of fact, all government is compulsion. The only question the nation has to consider is this, does the end justify the means? Is the freeing of the country from small-pox worth the price it is proposed to pay for it? If the answer were left to us—and we speak as experts—we should say the purchase is worth the price to be paid for it a hundred times over. Vaccination in an individual compared with small-pox in an individual is the merest trifle, not worthy of being put into comparison with the other. The vaccination of a whole community is hardly an inconvenience; but a plague of small-pox ravaging the people high and low is a tragedy and a calamity, the effect of which may not be entirely wiped out in a whole generation of the nation's life. It is infinitely more deadly, and to families infinitely more costly than a great war. Moreover, when and where does it cease? Without vaccination there is nothing to prevent any nation being visited by such a calamity as a small-pox epidemic every ten years.

The democracy has made up its mind to compulsory education—for a sufficient reason; it has made up its mind to an income-tax, to a costly navy, to a world-wide empire and its attendant cost in men and money—for sufficient reasons. It will make up its mind to compulsory re-vaccination if it can be shown a sufficient reason. We are convinced that it can be shown such a reason. The cases of Germany and the other continental countries are of themselves sufficient. The countries of Europe during the last twenty years have vaccinated with varying degrees of efficiency. The result is uniform throughout. This is the rule to-day for all Europe—the more efficient the vaccination the less deadly the small-pox. Germany, which has carried out both vaccination and re-vaccination, alike in her army and in her civil population, has beaten the European record in thoroughness for the past twenty years. She has also beaten the record in results; for, to-day, the German Empire is almost entirely free from small-pox. These are facts not to be reasoned away. For our part, we have always done our utmost to look at the question from the standpoint of the unlearned and the unconvinced; because our desire is, above all things, to do justice and to preserve liberty in the land. But now, looking at the question from this very standpoint of the unlearned, we feel that they can plead ignorance no more, and that the unconvinced have had all their arguments answered and their doubts removed. The democracy itself, if it is at all either generous or wise, will now rise up in a spirit of convinced commonsense and resolve to protect itself against one of the deadliest of all known scourges by the only, but most efficient, weapon which science has put into its hands.

